# Effects of Reticuloendotheliosis Virus Infection on Cytokine Production in SPF Chickens

**DOI:** 10.1371/journal.pone.0083918

**Published:** 2013-12-16

**Authors:** Mei Xue, Xingming Shi, Yan Zhao, Hongyu Cui, Shunlei Hu, Xianlan Cui, Yunfeng Wang

**Affiliations:** 1 Division of Avian Infectious Diseases, State Key Laboratory of Veterinary Biotechnology, Harbin Veterinary Research Institute, The Chinese Academy of Agricultural Sciences, Harbin, China; 2 National Engineering Research Center of Veterinary Biologics, Harbin, China; 3 Animal Health Laboratory, Department of Primary Industries, Parks, Water and Environment, Tasmania, Australia; Midwestern University, United States of America

## Abstract

Infection with reticuloendotheliosis virus (REV), a gammaretrovirus in the *Retroviridae* family, can result in immunosuppression and subsequent increased susceptibility to secondary infections. The effects of REV infection on expression of mRNA for cytokine genes in chickens have not been completely elucidated. In this study, using multiplex branched DNA (bDNA) technology, we identified molecular mediators that participated in the regulation of the immune response during REV infection in chickens. Cytokine and chemokine mRNA expression levels were evaluated in the peripheral blood mononuclear cells (PBMCs). Expression levels of interleukin (IL)-4, IL-10, IL-13 and tumor necrosis factor (TNF)-α were significantly up-regulated while interferon (IFN)-α, IFN-β, IFN-γ, IL-1β，IL-2, IL-3, IL-15, IL-17F, IL-18 and colony-stimulating factor (CSF)-1 were markedly decreased in PBMCs at all stages of infection. Compared with controls, REV infected chickens showed greater expression levels of IL-8 in PBMCs 21 and 28 days post infection. In addition, REV regulates host immunity as a suppressor of T cell proliferative responses. The results in this study will help us to understand the host immune response to virus pathogens.

## Introduction

Reticuloendotheliosis viruses (REVs) are a group of viruses in the family Retroviridae, speciﬁcally gammaretroviruses in the same genus as mammalian C-type retroviruses [1]. The REV group includes defective REV-T [2,3], non-defective REV-A [4,5], chick syncytial virus[6], duck infectious anemia virus [7] and spleen necrosis virus (SNV) [8]. The non-defective REV-A virus has a 8.7-kb genome consisting of a group-specific antigen (gag), polymerase (pol) and envelope (env) genes flanked by long-terminal repeats (LTRs) [9].

REVs cause immunosuppression, runting disease, and lymphoma in a variety of avian hosts that include chickens, turkeys, ducks, geese, pheasants, peafowl, and some other bird species [10]. Some studies have shown that REVs are important cofactors for a number of avian diseases [11–13]. In addition, REV infection has also been associated with poor immune responses to chicken vaccines [14]. The enhancement of these diseases by concomitant REV infection is the most likely consequence of immunosuppression, but the mechanism of REV-induced immunosuppression has not been completely characterized.

Cytokines play a key role in the innate immune system [15]. Most cytokines have pleiotropic or redundant functions, and the level of one cytokine is tightly regulated by other cytokines. For example, an increase in Th2 cytokines (e.g. IL-4 and IL-10) can result a decrease in Th1 cytokines (e.g. IFN-γ and IL-2) [16]. Therefore, it is important to examine multiple cytokines in response to REV infection to understand the roles of cytokines in viral pathogenicity. To date, several studies have focused on the effects of REV on only a few pro-inﬂammatory cytokines [17,18]. Other important pro-inﬂammatory cytokines, anti-inﬂammatory cytokines, and chemokines that have been associated with other retrovirus infections and pathogenicity have not been studied [19,20].

The bDNA assay, a sandwich nucleic acid hybridization platform in which targets are captured through cooperative hybridization of multiple probes, detects RNA directly, without either a reverse transcription step or polymerase chain reaction process. This assay provides a powerful method to obtain reliable measurements of multiple-gene expressions and ensures high assay specificity [21].

The main aims of this study were: 1) to determine the effect of REV-A infection on expression of mRNA for Th1-related cytokines (IFN-γ, IL-2, IL-15 and IL-18), Th2-related cytokines (IL-4, IL-10 and IL-13), other cytokines (IL-1β, IL-3, IL-17F, IFN-α, IFN-β, TNF-α, and CSF-1) and chemokine IL-8, in speciﬁc pathogen free (SPF) White Leghorn chickens; 2) to determine the effect of REV-A infection on T cell proliferation and the balance of CD4+/CD8+.

## Materials and Methods

### Ethics Statement

Care of laboratory animals and animal experimentation were conducted following ‘‘the Australian National Health and Medical Research Council’s Australian Code of Practice for the Care and Use of Animals for Scientific Purposes’’ guidelines for housing and care of laboratory animals. All animal studies were approved by the Animal Ethics Committee of Harbin Veterinary Research Institute of the Chinese Academy of Agricultural Sciences (SYXK (Hei) 2011022).

### Experimental animals and infection virus strain

All the chickens used in this experiment were one-day-old SPF White Leghorn chickens obtained from Harbin Veterinary Research Institute, The Chinese Academy of Agricultural Sciences. Chickens were kept in isolators at Harbin Veterinary Research Institute throughout the experiment. 

Chickens were infected with the HLJ07I strain of REV-A (GenBank accession no. GQ375848) that was isolated from Heilongjiang Province of China in 2007. REV was propagated in chicken embryo fibroblast (CEF) as previously described [22].

### Experimental design

Forty one-day-old SPF chickens were randomly divided into two groups and were housed in the isolators. One group of chickens (n = 20) was inoculated intra-abdominally with 10^4.6^ tissue culture infective doses 50% (TCID_50_) of the REV-A HLJ07I strain on day 3 of age. The rest (n = 20) were kept as uninfected controls. Infected and uninfected control chickens were kept in separate isolators with similar environmental conditions. On 7, 14, 21 and 28 days post infection (dpi), representing different stages of REV pathogenesis, five chickens were randomly selected from each group. Chicken peripheral blood mononuclear cells (PBMCs) were isolated from whole blood over a discontinuous density gradient of Ficoll-Histopaque (density = 1.077 g/ml), washed twice in PBS, and the number of viable cells was determined by an automatic cell counter (NucleoCounter, NC-100, Chemometech, Denmark). At the end of the experiment, chickens were anesthetized by CO_2_ inhalation and euthanized by cervical dislocation.

### Qualitative RT-PCR assay

The viral RNA copy numbers in the PBMCs were determined by quantitative real-time RT-PCR. RNA was extracted from PBMCs using TRIzol© (Invitrogen, Carlsbad, CA, USA) according to the manufacturer’s instructions and samples were subsequently subjected to DNase treatment (Invitrogen, Carlsbad, CA, USA). Total RNA in each sample was measured using a Spectrophotometer (NanoVue, GE Healthcare, Uppsala, Sweden). cDNA was synthesized in a final volume of 30 µL containing 6 µL of 5×AMV buffer, 0.5 µL of avian myeloblastosis virus (AMV) reverse transcriptase (Takara, Shiga, Japan), 2 µL of oligo (dT), 2 µL of 10 mmol/L deoxynucleotide triphosphate mix, 1 µL of Cloned Ribonuclease Inhibitor, 3.5 µL of RNase-free water, and 5 µL of total RNA. The reaction was done at 25°C for 10 min and 42°C for 60 min. The synthesized cDNA was stored at −20°C until used in the real-time PCR. The absolute REV genome load in the REV-infected chicken’s PBMCs was quantified using primers specific for REV-gag gene. The primers used were: forward primer (5' AGACTCGCATTGTCGATGTCTTG 3') and reverse primer (5' CAAATCTTTGCCAATCAA TATCAG 3'). Linear regression analysis of the standard curve was used to estimate the number of viral genomic RNA copies. The standard RNA curve was linear in the range between 10^2^ molecules at the lower limit and 10^9^ molecules at the upper limit. A real time-PCR assay was performed in a total volume of 20 µL containing 10 µL of SYBR^®^ Premix Ex Taq^TM^ (2×; Takara, Shiga, Japan), 100 ng of cDNA, 10 pmol of forward primer, and 10 pmol of reverse primer using a LightCycler® 480 Real-Time PCR System (Roche Diagnostics). The PCR protocol consisted of an initial denaturation step at 95°C for 120 s and 40 cycles of denaturation (95°C for 15 s), annealing (61°C for 30 s) and extension (72°C for 15 s). For each step, the temperature transition rate was 20°C/s. Experiments on each sample were performed in triplicate with the above primers. The formula used to quantify the relative amount of gene expression was 2^−ΔCT^. The absolute numbers of REV genome per 10^6^ cells were calculated based on the standard curve.

### Measurement of cytokine and chemokine expression by bDNA assay

In this study, speciﬁc oligonucleotide probe sets for target genes (Table 1) for use in QuantiGene Plex 2.0 Reagent System (Affymetrix Inc., Santa Clara, California, USA) were designed by standard probe design software. bDNA analysis was performed using the reagents provided by the manufacturer (Affymetrix Inc., Santa Clara, California, USA) in a three-step procedure, which included specimen preparation, hybridization, and detection. Brieﬂy, PBMCs from treated (infected with REV-A HLJ07I strain) or untreated chickens in microfuge tubes were mixed with 80 µL of lysis mixture provided by QuantiGene Sample Processing Kit (Affymetrix Inc., Santa Clara, California, USA) and incubated at 50°C for 1 h to release mRNA. Aliquots of 80 µL of lysate were transferred to capture plates, which contained 20 µL of pooled speciﬁc probes, and were incubated for hybridization with the probes for 20 h at 54°C. The hybridization mixtures were removed, and microspheres in the capture plates were washed three times with wash buffer to remove unbound material. For signal amplification and hybridization, a volume of 100 µL of 2.0 Pre-Amplifier Working Reagent (Affymetrix Inc., Santa Clara, California, USA) was added to each well, and the plates were incubated at 50°C for 1 h. After washing three times with wash buffer, a volume of 100 µL of Ampliﬁer Working Reagent (Affymetrix Inc., Santa Clara, California, USA) was added to each well, and the plates were incubated at 50°C for 1 h. Plates were then washed three times as described above and incubated with 100 µL of Label Probe Working Reagent (Affymetrix Inc., Santa Clara, California, USA) at 50°C for 1 h. These plates were washed again three times, and 100 µL of the ﬂuorescent reagent SAPE Working Reagent (Affymetrix Inc., Santa Clara, California, USA) was added to each well. The ﬂuorescence intensity of SAPE was proportional to the amount of mRNA transcript captured by the microspheres in each well. The amount of multiple-target mRNAs in each sample was simultaneously determined by measuring the wavelengths of color-coded microspheres and the intensities of the luminescent emission of SAPE using a Luminex 200 (Molecular Devices).

**Table 1:** Regions of target genes that were used to design bDNA probe sets for the ampliﬁcation of the mRNAs of 15 cytokines/chemokines and the internal control.

	Names	Sequence length	Probe set region	GenBank Accession Number
Cytokines	IFN-α	767	19-431	NM_205427
	IFN-β	612	67-528	NM_001024836
	IFN-γ	1330	193-856	NM_205149
	IL-1β	1107	351-709	NM_204524
	IL-2	798	75-705	NM_204153
	IL-15	857	77-718	NM_204571
	IL-17F	935	2-522	NM_204460
	IL-18	724	2-590	NM_204608
	IL-3	417	3-388	NM_001007083
	Csf1	813	17-534	NM_001193295
	IL-4	411	11-362	NM_001007079
	IL-10	528	39-394	NM_001004414
	IL-13	417	10-370	NM_001007085
	TNF-α	740	37-479	NM_204267
Chemokine	IL-8	1182	25-650	NM_204608
Internal control	GAPDH	1288	269-503	NM_204305

All data were analyzed using Luminex IS 2.3 program. An acquisition gate between 5000 and 20,000 was set to exclude any doublet events and ensure that only single microspheres were measured. One hundred events per region were collected. For all samples in the bDNA assay, the background signal was determined in the absence of target mRNAs and was subtracted from the signal obtained in the presence of target mRNAs. The expression levels of the cytokines and chemokines were normalized to GAPDH. Three independent experiments were performed for each treatment.

### Cell proliferation assay

PBMCs were isolated from heparinized peripheral blood of REV-A infected chickens or controls using Ficoll-Histopaque gradient centrifugation method and labeled with 2.5 µM CFSE (5-carboxyﬂuorescin diacetate succinimidyl ester, Invitrogen, Carlsbad, CA). Brieﬂy, PBMCs (1 ×10^7^ cells/ml) were incubated at 37°C for 10 min with 2.5 µM CFSE and 5 ml of culture medium (10% FBS) was added to stop CFSE cell staining. Cells were washed three times in PBS and resuspended at a concentration of 1×10^7^ cells/ml in culture medium. Stained cells were cultured in 96-well flat bottom plate (4 × 10^5^ cells/well). The temperature, time and pH of the incubations were optimized. PBMCs stimulated with 1 µg/well of Concanavalin A (ConA; Sigma, St. Louis, MO) were used as the positive control. Cells cultured in media containing PBS were used as the negative control. After incubating at 37°C with 5% CO_2_ for 4 days, CFSE-labeled PBMCs were incubated with a mouse monoclonal antibody, R-PE-anti-chicken CD3 (Southern Biotech, Birmingham, AL) at room temperature for 30 min. After removal of the antibody and washing three times, cells from each treatment group were fixed with 4% paraformaldehyde and analyzed using a Beckman FC500 ﬂow cytometer. 

### Flow cytometric analysis

The PBMCs were collected to detect the diversity of CD4+/CD8+. Briefly, 1×10^6^ PBMCs were incubated with mouse anti-chicken CD3-SPRD, anti-chicken CD4-FITC, and anti-chicken CD8-RPE (Southern Biotech, Birmingham, AL) at 4°C for 30 min. The cells were washed three times with PBS containing 1% fetal bovine serum. The cells were then suspended with PBS and analyzed by the FacsCalibur and CellQuest software (Becton Dickinson, Franklin Lakes, NJ). The viable lymphocytes were calculated on the basis of forward and sideward scatter characteristics, and 20,000 events were analyzed for positive staining with SPRD, FITC, and RPE antibodies.

### Statistical Analysis

Statistical analyses of signiﬁcance were performed using Student’s t-test. The results were considered statistically significant at the level of P < 0.05 or P < 0.01.

## Results

### Replication of the virus in PBMCs

**Figure 1:**
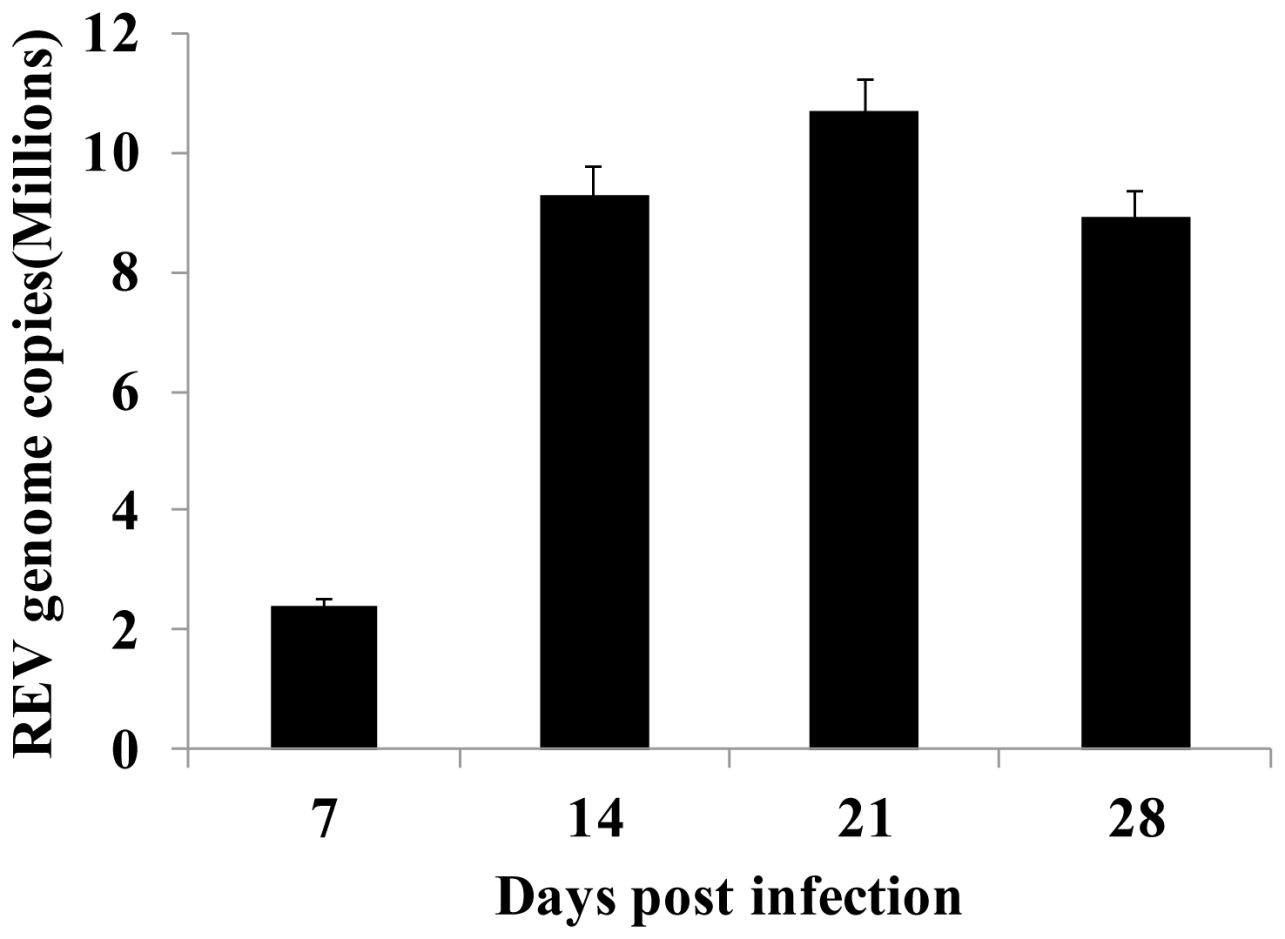
REV genome load in infected PBMCs. Chickens were infected with the HLJ07I strain of REV and sampled at 7, 14, 21 and 28 days post-infection. Gag copy numbers in 10^6^ PBMCs were quantitated using real-time RT-PCR. At least three samples were analyzed in duplicate at each sampling time point. The error bars represent standard error of the mean.

To assess the replication ability of the REV HLJ07I strain in the PBMCs, the RNA genome copy numbers for the virus were detected using real-time RT-PCR at 7, 14, 21, and 28 days after infection. As shown in Figure 1, REV genome was ﬁrst detected at 7 dpi, increasing rapidly from day 7 and peaked at day 21. Thereafter, REV genome copy number steadily declined until the termination of the experiment at 28 dpi.

### Kinetics of cytokine and chemokine mRNA expression after infection with the REV-A HLJ07I strain

The reproducibility of the bDNA assay was evaluated by analyzing the expression levels of 14 cytokines (IL-1β, IL-2, IL-3, IL-4, IL-10, IL-13, IL-15, IL-17F, IL-18, IFN-α, IFN-β, IFN-γ, CSF, and TNF-α) and one chemokine (IL-8) in duplicate in uninfected PBMCs. Three independent experiments were performed using PBMCs. The mRNA level of GAPDH was measured as an internal control. The average values of duplicate wells were used to calculate the coefﬁcient of variation (CV) of the background expression level of these 15 cytokines and chemokine in three independent experiments. As shown in Table 2, the CV values of all of the cytokine and chemokine mRNA levels with or without normalization to GAPDH were less than 5%, which indicated a high reproducibility of the measurements. These results demonstrated that the bDNA assay was a reliable method to simultaneously evaluate the expression levels of multiple cytokines and chemokines.

**Table 2:** Reproducibility of the bDNA multiplex assay and the effects of GAPDH normalization.

		Unnormalized mRNA levels			mRNA levels normalized with GAPDH		
		Mean^a^	SD^b^	CV^c^ (%)	Mean	SD	CV (%)
**Cytokines**	IFN-α	503.86	10.72	2	0.118	0.0041	3
	IFN-β	597.70	11.81	2	0.139	0.0057	4
	IFN-γ	9505.47	83.24	1	1.901	0.0401	2
	IL-1β	1964.67	12.43	1	0.43	0.0122	3
	IL-2	217.78	10.91	5	0.053	0.0025	5
	IL-15	710.48	28.93	4	0.16	0.0046	3
	IL-17F	1827.52	56.23	3	0.389	0.0132	3
	IL-18	9299.72	110.45	1	1.927	0.0419	2
	IL-3	3060.02	61.23	2	0.702	0.0164	2
	Csf1	3043.08	40.45	1	0.642	0.0132	2
	IL-4	8100.37	90.76	1	1.718	0.0389	2
	IL-10	5605.61	76.54	1	1.144	0.0343	3
	IL-13	5317.56	85.63	2	1.103	0.0389	4
	TNF-α	469.17	23.57	5	0.09	0.0032	4
**Chemokine**	IL-8	10515.62	139.98	1	2.391	0.0517	2

^a^ The average values of three independent experiments using PBMCs prepared from the same chicken. Each value was calculated from the expression levels of duplicate wells. The mRNA level of GAPDH was measured as an internal control of baseline expression level.

^b^ Standard deviation of values from three independent experiments.

^c^ The coefﬁcient of variation (CV) was calculated by the formula: CV= SD/mean×100%.

**Figure 2:**
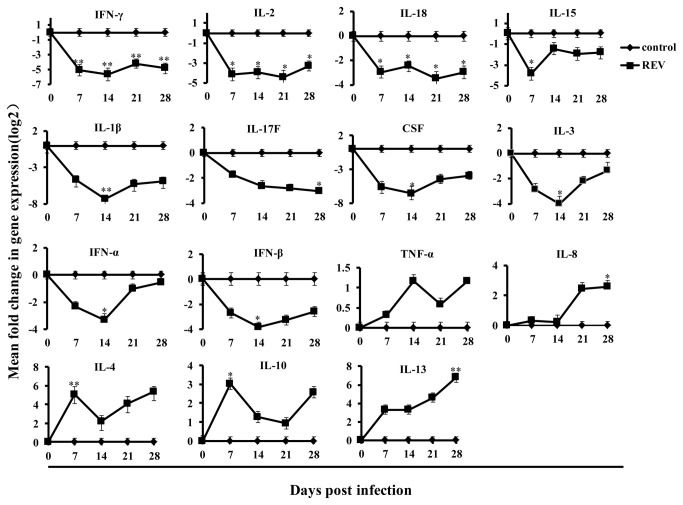
The relative cytokine and chemokine mRNA levels in PBMCs of chickens infected with REV-A strain HLJ07I or uninfected controls for 7, 14, 21 and 28 days. Values were normalized to the endogenous GAPDH control and were presented as the log_2_ mean fold-change in mRNA expression (relative to the uninfected control). Data are the means of three independent experiments. * indicates P < 0.05 and ** indicates P < 0.01 when the REV-infected group was compared with the control group.

To determine the effects of REV infection on cytokine and chemokine mRNA expression, transcript levels of 14 cytokines (IL-1β, IL-2, IL-3, IL-4, IL-10, IL-13, IL-15, IL-17F, IL-18, IFN-α, IFN-β, IFN-γ, CSF, and TNF-α) and one chemokine (IL-8) were examined. At 7, 14, 21, and 28 days after infection with REV-A HLJ07I strain, PBMCs were isolated and detected by bDNA assay. As shown in Figure 2, the expression of pro-inflammatory cytokines IFN-α, IL-1β and IL-17F were down-regulated in chickens after REV infection compared to uninfected chickens. Analysis of Th1 and its regulatory cytokines IFN-γ, IL-2, IL-15 and IL-18 revealed that the levels of these cytokines were decreased following REV infection. After REV infection, the expression levels of Th2 and regulatory cytokines (IL-4、IL-10 and IL-13) were drastically increased after 7 dpi, and moderately increased at 14 days and then up-regulated after 21 and 28 days following infection. The production of TNF-α was markedly up-regulated after 7 and 14 dpi. The levels of IFN-β, IL-3 and CSF were signiﬁcantly decreased at 7 and 14 days after infection and then moderately increased at 21 and 28 dpi. We analyzed the IL-8 expression and no significant changes were observed at 7 and 14 days post infection, while the expression levels of IL-8 were gradually up-regulated after 21 and 28 dpi.

### Inhibition of T cell functions by REV infection

**Figure 3:**
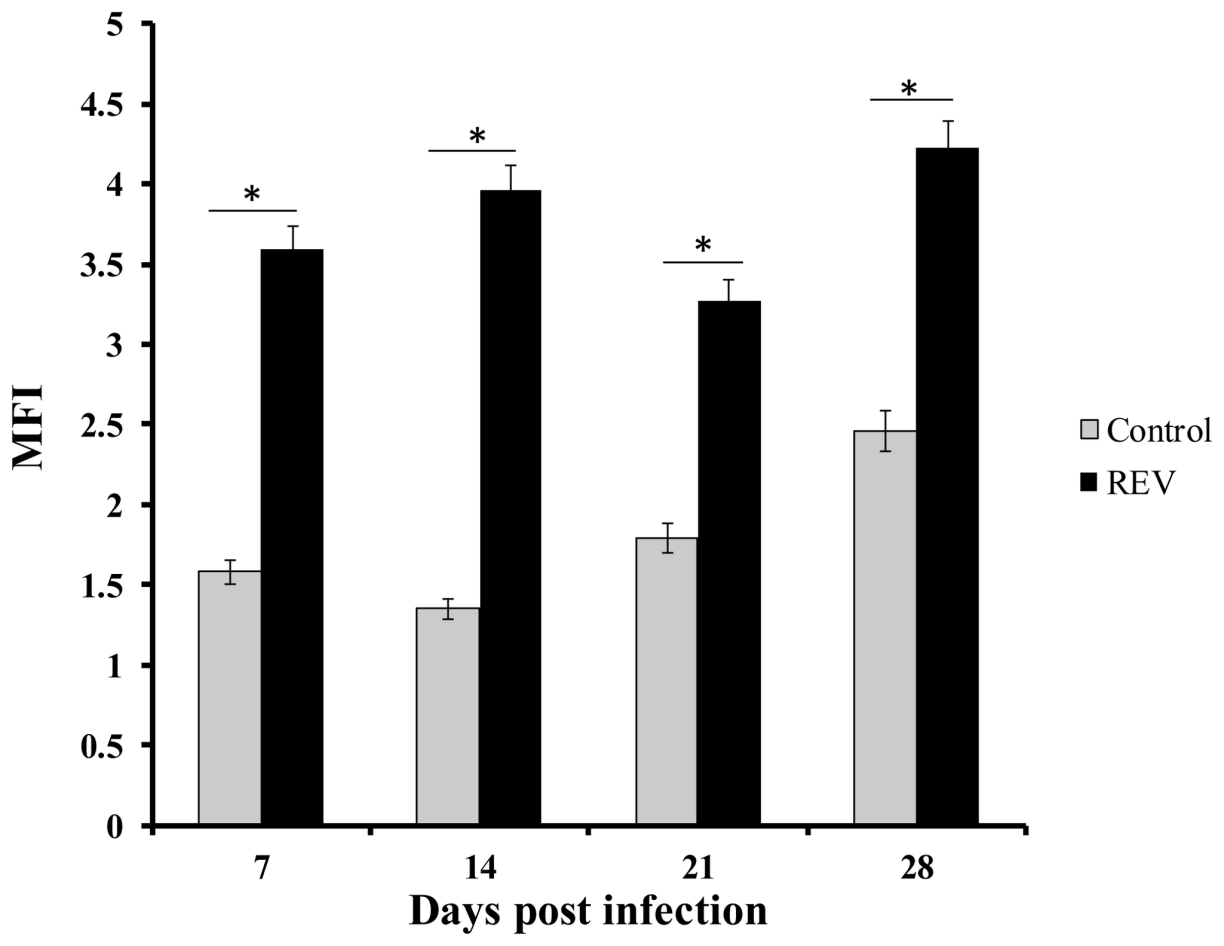
The proliferation of PBMCs post infection of REV. The PBMCs (1 ×10^7^ cells/ml) were isolated from heparinized peripheral blood of REV-A infected or uninfected control chickens. The mean fluorescence intensity (MFI) was statistically analyzed. * indicates P < 0.05 when the REV-infected group was compared with the control group.

To explore the functions of REV infection in chicken immune response, we assessed the effect of REV-A on PBMC proliferation. As shown in Figure 3, the rate of PBMC proliferation in chickens infected with REV-A was signiﬁcantly decreased compared to that of uninfected chickens (p < 0.05). 

**Figure 4:**
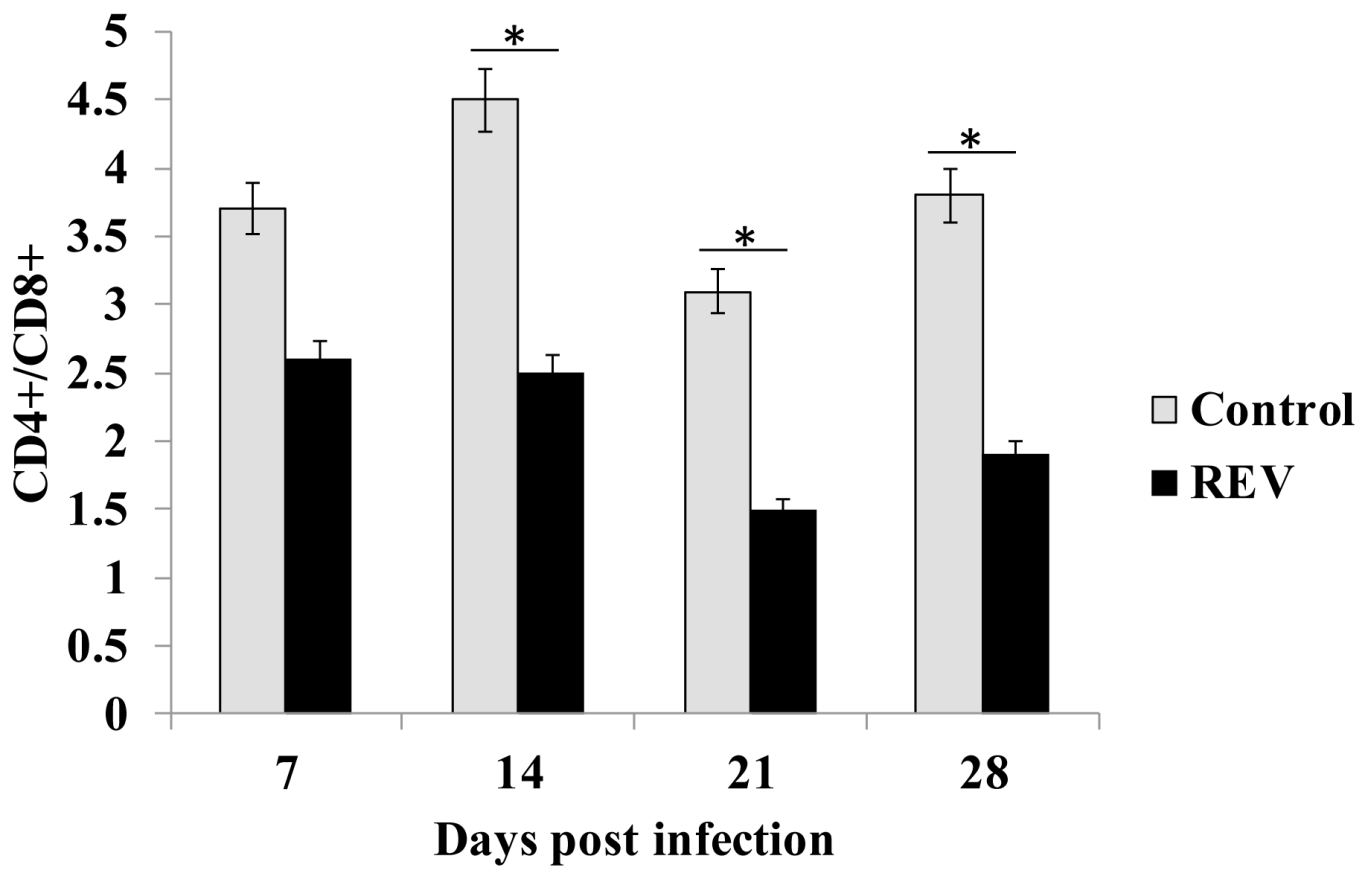
The subpopulation ratios of CD4+/CD8+ in the PBMCs of chickens infected with REV detected by flow cytometry. The PBMCs were isolated from the heparinized peripheral blood and stained with mouse monoclonal antibodies against chicken CD3, CD4, and CD8. CD4+/CD8+ ratios were calculated from the number of cells labeled with the ﬂuorescent monoclonal antibodies of anti-CD4 or anti-CD8 analyzed using a ﬂow cytometer. All data were expressed as mean ± standard error. * indicates P < 0.05 when the ratio of the REV-infected group was compared with that of the control group.

CD4+/CD8+ ratios were calculated from the number of cells labeled with the ﬂuorescent monoclonal antibodies of anti-CD4 or anti-CD8 analyzed using a ﬂow cytometer. As shown in Figure 4, the ratios of CD4+/CD8+ in chickens infected with REV-A were lower than those of uninfected chickens. 

## Discussion

In this study the kinetic responses of immune-related cytokines and chemokines in PBMCs were examined following experimental infections of chickens with REV-A. Cytokines and chemokines play important roles in regulating innate immune responses and acquired immunity [15]. Analysis of cytokine and chemokine proﬁles will provide more information on the mechanism of immunosuppression caused by REV. In comparison to the cases with humans or mice, the detection of avian cytokines and chemokines has been hampered by the lack of speciﬁc antibodies and reliable bioassays [23]. Up to now, there are no comprehensive studies on the role of cytokines or chemokines during *in vivo* REV-A infections of the chickens. In this study, expression levels of IL-4, IL-10, IL-13 and TNF-α were significantly up-regulated while the expression levels of IL-1β，IL-18，IFN-γ, IL-2, IL-15, IL-17F, IFN-α, IFN-β, IL-3 and CSF-1 were markedly decreased in PBMCs at all stages of infection. Compared with controls, REV infected chickens showed greater expression levels of IL-8 in PBMCs 21 and 28 dpi. The expression of Th1-related cytokines IFN-γ, IL-2, IL-15 and IL-18 were down-regulated, while the production of Th2-related cytokines IL-4, IL-10 and IL-13 were drastically increased, which showed a trend for Th1 to Th2 conversion. PBMC proliferation assay showed that REV inhibited proliferative responses in chicken lymphocytes stimulated with T helper mitogen ConA. Furthermore, the chickens infected with REV induced a lower ratio of CD4+/CD8+. These data suggested that REVs have immunosuppressive effects in chickens.

One set of molecules involved in the immune response is Th2 cytokines. In mammals, it has been known for some time that the balance between Th1/Th2 lymphocyte subsets determines susceptibility to some disease states [24]. Thus, an unusually dominant Th1 response is often associated with autoimmunity, while improper development of Th2 immunity can lead to allergic diseases [25]. Th2 cells are necessary for inducing the humoral response to combat virus infection [26]. As in mammals, the chicken genome contains a cluster of Th2 cytokine genes including IL-10, IL-4 and IL-13, all of which are expressed in lymphoid tissues [27]. IL-10 plays an important role in regulating immune responses and inhibiting the synthesis of inflammatory and pro-inﬂammatory cytokines (such as IL-1β), thus down-regulating inﬂammatory Th1 responses [28]. IL-10 is highly regarded as an immunosuppressive cytokine and plays a role as the mediator of tumor regression [29]. It has been reported that IL-10 can be secreted by tumor cells [30] and high IL-10 levels were observed in areas of spontaneous regression of primary melanoma [31]. The predominant expression of immunosuppressive cytokines in cancer patients induces an immunosuppressive state in the immunological microenvironment of the tumor [29] and enables tumor to escape from immune recognition [32,33]. Since low levels of IL-10 increase resistance and high levels increase susceptibility [34], the high expression of IL-10 may help us to understand the increase in susceptibility to concurrent or secondary bacterial or viral infections post REV-A infection. IL-4 plays an important role in the differentiation of naive T cells toward a Th2 phenotype. IL-13 is structurally similar to IL-4 and is produced by Th2 CD4+ T cells as well as epithelial cells. IL-10, IL-4 and IL-13 are markedly increased in earlier days and later days of REV-A infection. It is associated with down-regulating inﬂammatory (Th1) response and results in driving Th2 cell development. In HIV, it has been reported that viral infection induced FcepsilonRI+ hematopoietic cells to produce IL-4, which inactivated the host adaptive immune response [35]. In addition, the conversion from a Th1 response to a Th2 response during viral infection favors infection and the spread of HIV-1 [36].

Another set of molecules involved in the immune response is Th1 cytokines. IL-2 and IL-15 are structurally homologous to Th1 or Th1 related cytokines produced by mononuclear phagocytes and other cell types in response to viral infection [37,38]. Both cytokines stimulate proliferation of chicken T lymphocytes and NK cells [38]. Interferons were ﬁrst described in chickens [39] and have been shown to have various immunomodulating effects on a wide variety of tissues and combat the replication of the viruses in the host cells. The decreased levels of IL-2, IL-15, IFNα, IFNβ and IFN-γ indicated that infection of REV-A suppressed Th1-type immune response. It has been reported that the expression level of immunosuppressive cytokines, such as IL-10, was significantly higher than that of immunostimulatory cytokines, such as IL-2 and IFN-γ, in various cancer patients [40,41]. IFN-γ production during REV infection has been examined by quantitative RT-PCR [17] and by antigen capture ELISA [18]. Infection with REV-CS resulted in a 10-fold increase in IFN-γ mRNA levels in 9- to 10- or 30-day-old birds [17]. Chickens infected with SNV strain of REV at 5 days of age showed 3 to 5 fold increased level of IFN-γ between 7 and 28 dpi as measured by Ag capture ELISA [18]. It remains to be determined if the difference between the cytokine responses induced by these two REV strains is related to their pathogenicity, antigenicity, and/or other unknown factors. 

IL-1β is a powerful pro-inﬂammatory cytokine secreted by many different types of cells, with stimulated macrophages being the major producer. In this study IL-1β was down-regulated after REV infection when bDNA technology was utilized for testing. No significant changes in the level of IL-1β transcripts was observed at either 7 or 14 days post REV-CS strain infection [17], which was different from the results in this study. In another study, infection with MDV JM-16 strain could not induce IL-1β expression in the spleen but RK-1 strain led to induction of IL-1β at 4 dpi in both N2a and P2a chicken lines [42]. IL-18 is structurally homologous to IL-1β and plays important roles in initiating inﬂammation. In mammals, IL-18 is an inducer of cell-mediated immunity, especially in combination with IL-12, and is primarily associated in Th1 responses to intracellular pathogen infections [43]. IL-18 transcription was down-regulated between 7 and 28 dpi post REV-A infection. IL-17 is produced by activated memory T cells and, like IL- 1β, induces the production of other pro-inﬂammatory cytokines, such as TNF-α and IL-1β. The results in this study demonstrated that IL-17F transcript was gradually down-regulated following REV-A infection. Thus, the decreased levels of transcripts for several chicken pro-inﬂammatory cytokines in this study suggest that these molecules may be relevant to the tumor and immune-suppression caused by REV infection.

The CSFs are a family of polypeptide growth factors critical to the development of haematopoietic cells, including those of the monocyte/macrophage lineage [44]. CSF-1 is an important mediator of inflammation, and also represents a key element of a possible haematopoietic growth factor network active at sites of inflammation [45]. For example, in the inflamed joint CSF-1 may drive the differentiation and activation of monocytes/macrophages, causing the release of inflammatory mediators and resulting in inflammation and local tissue damage. Compared with mammalian IL-3, chicken IL-3 was reported to be expressed at higher levels in all tissues [27], which concurs with our mRNA expression data. In this study, CSF-1 and IL-3 were significantly down-regulated at 7 and 14 dpi, and gradually increased at 21 and 28 dpi. The down-regulated expression of CSF-1 and IL-3 were consistent with other pro-inﬂammatory cytokines and demonstrated the immunosuppressive state after REV-A infection.

TNF-α is a potent immunomodulator and proinflammatory cytokine that has been implicated in the pathogenesis of autoimmune and infectious diseases. It has been reported that TNF-α system was activated during HIV-1 infection and the raised levels increased with disease progression and degree of immunodeficiency [46]. Since TNF-α has a strong antitumoral action [47], the up-regulated expression of TNF-α post REV-A infection may correlate with tumor caused by REV. 

Chemokines are another group of regulators of immunity that has important roles in disease etiopathology and the immune response after viral infection [48]. IL-8 is a member of the chemokines, which is known as an important mediator of inﬂammation that recruits and activates leukocytes to sites of infection [49,50]. In addition, the potential role of IL-8 in viral infections of chickens was also indicated. For examples, large increases in IL-8 mRNA were seen in the brains after Marek’s disease virus infection [42] or in the chicken macrophages exposed to infectious bursal disease virus [51]. Moreover, human and murine tumors also frequently secrete IL-8 [52]. IL-8 was up-regulated in the late stage of infection in this study, and the over-expression of IL-8 would induce excessive accumulation of lymphocytes and mononuclear cells in infected tissues and cause cytokine imbalances.

There are a number of possible explanations as to why IL-1β, IL-2 and IFN-γ expression levels presented in this study were different from the previous study [17]. Firstly, the virus strains and chicken lines used in both studies were different. Secondly, the age of infection differed in the two studies, with chickens being infected at 9-30 days of age in the report by Schat et al. [17] and chickens being infected at 3 days of age in this study. Thirdly, the total amount of RNA used in the test assays might be different in each report. In this study, 10^6^ PBMC RNA was used in each reaction while the actual amount of input RNA used in the reaction by Schat et al. [17] was unknown. Thus, if less RNA is added to each reaction, a sample may be negative for a specific cytokine at that level of sensitivity, but may be positive if more input RNA is added. Finally, the methodology used to test cytokines was different in these two studies. bDNA assay was used in this study compared to qualitative RT-PCR assay used in the previous report [17]. The bDNA assay is a sandwich nucleic acid hybridization platform in which target-specific RNA molecules are captured through cooperative hybridization of multiple probes. It has been demonstrated that this assay enables the reliable detection and quantitation of multiple-gene expressions simultaneously [53].

Our data demonstrated that T cell proliferative responses were decreased and the ratio of CD4+/CD8+ was lower in REV infected chickens. The inhibition of T-cell proliferation and the lower ratio of CD4+/CD8+ induced by REV would enable the virus to downregulate the host immune response, thereby compromising the ability of the host to develop effective protective immunity to other pathogens.

To the best of our knowledge, this is the ﬁrst comprehensive study of differential cytokine and chemokine expression in PBMCs infected with REV-A strains using bDNA multiple measurement technology. Based on the results in this study, REV infection causes disruption of cytokine networks, inhibits chicken lymphocyte proliferation, enhances the immunosuppressive effect, and thus increases susceptibility to concurrent or secondary bacterial or viral infections and results in poor immune responses to chicken vaccines. Further investigations are required to evaluate the effect of differential expression of these cytokines and chemokines on the tumors and immune response of viral infection.

## References

[1] Coffin JM (1996) Retrovirus restriction revealed. Nature 382: 762–763. doi:10.1038/382762a0. PubMed: 8752269. 8752269

[2] Hoelzer JD, Franklin RB, Bose HR Jr. (1979) Transformation by reticuloendotheliosis virus: development of a focus assay and isolation of a nontransforming virus. Virology 93: 20–30. doi:10.1016/0042-6822(79)90272-1. PubMed: 219596. 219596

[3] Hoelzer JD, Lewis RB, Wasmuth CR, Bose HR Jr. (1980) Hematopoietic cell transformation by reticuloendotheliosis virus: characterization of the genetic defect. Virology 100: 462–474. doi:10.1016/0042-6822(80)90536-X. PubMed: 6243436. 6243436

[4] Witter RL, Smith EJ, Crittenden LB (1981) Tolerance, viral shedding, and neoplasia in chickens infected with non-defective reticuloendotheliosis viruses. Avian Dis 25: 374–394. doi:10.2307/1589930. PubMed: 6266388. 6266388

[5] Chen PY, Cui Z, Lee LF, Witter RL (1987) Serologic differences among nondefective reticuloendotheliosis viruses. Arch Virol 93: 233–245. doi:10.1007/BF01310977. PubMed: 3030238. 3030238

[6] Cook MK (1969) Cultivation of a filterable agent associated with Marek's disease. J Natl Cancer Inst 43: 203–212. PubMed: 4307703. 4307703

[7] Ludford CG, Purchase HG, Cox HW (1972) Duck infectious anemia virus associated with Plasmodium lophurae. Exp Parasitol 31: 29–38. doi:10.1016/0014-4894(72)90044-6. PubMed: 4622046. 4622046

[8] Trager W (1959) A new virus of ducks interfering with development of malaria parasite (Plasmodium lophurae). Proc Soc Exp Biol Med 101: 578–582. doi:10.3181/00379727-101-25023. PubMed: 13675324. 13675324

[9] Beemon KL, Faras AJ, Hasse AT, Duesberg PH, Maisel JE (1976) Genomic complexities of murine leukemia and sarcoma, reticuloendotheliosis, and visna viruses. J Virol 17: 525–537. PubMed: 176429. 176429 10.1128/jvi.17.2.525-537.1976 PMC515444

[10] Bohls RL, Linares JA, Gross SL, Ferro PJ, Silvy NJ et al. (2006) Phylogenetic analyses indicate little variation among reticuloendotheliosis viruses infecting avian species, including the endangered Attwater's prairie chicken. Virus Res 119: 187–194. doi:10.1016/j.virusres.2006.01.011. PubMed: 16497405. 16497405

[11] Cui Z, Sun S, Zhang Z, Meng S (2009) Simultaneous endemic infections with subgroup J avian leukosis virus and reticuloendotheliosis virus in commercial and local breeds of chickens. Avian Pathol 38: 443–448. doi:10.1080/03079450903349188. PubMed: 19937533. 19937533

[12] Davidson I, Shkoda I, Perk S (2008) Integration of the reticuloendotheliosis virus envelope gene into the poultry fowlpox virus genome is not universal. J Gen Virol 89: 2456–2460. doi:10.1099/vir.0.2008/001313-0. PubMed: 18796713. 18796713

[13] Sun AJ, Xu XY, Petherbridge L, Zhao YG, Nair V et al. (2010) Functional evaluation of the role of reticuloendotheliosis virus long terminal repeat (LTR) integrated into the genome of a field strain of Marek's disease virus. Virology 397: 270–276. doi:10.1016/j.virol.2009.11.017. PubMed: 19962172. 19962172

[14] Kawamura H, Wakabayashi T, Yamaguchi S, Taniguchi T, Takayanagi N (1976) Inoculation experiment of Marek's disease vaccine contaminated with a reticuloendotheliosis virus. Natl Inst Anim Health Q (Tokyo) 16: 135–140. PubMed: 189219. 189219

[15] Belardelli F (1995) Role of interferons and other cytokines in the regulation of the immune response. APMIS 103: 161–179. doi:10.1111/j.1699-0463.1995.tb01092.x. PubMed: 7538771. 7538771

[16] Marzi M, Vigano A, Trabattoni D, Villa ML, Salvaggio A et al. (1996) Characterization of type 1 and type 2 cytokine production profile in physiologic and pathologic human pregnancy. Clin Exp Immunol 106: 127–133. PubMed: 8870710. 8870710 10.1046/j.1365-2249.1996.d01-809.x PMC2200555

[17] Markowski-Grimsrud CJ, Schat KA (2003) Infection with chicken anaemia virus impairs the generation of pathogen-specific cytotoxic T lymphocytes. Immunology 109: 283–294. doi:10.1046/j.1365-2567.2003.01643.x. PubMed: 12757624. 12757624 PMC1782969

[18] Zheng YS, Cui ZZ, Zhao P, Li HM, Liu CY et al. (2007) Effects of reticuloendotheliosis virus and Marek's disease virus infection and co-infection on IFN-gamma production in SPF chickens. J Vet Med Sci 69: 213–216. doi:10.1292/jvms.69.213. PubMed: 17339769. 17339769

[19] Peterson KE, Robertson SJ, Portis JL, Chesebro B (2001) Differences in cytokine and chemokine responses during neurological disease induced by polytropic murine retroviruses Map to separate regions of the viral envelope gene. J Virol 75: 2848–2856. doi:10.1128/JVI.75.6.2848-2856.2001. PubMed: 11222710. 11222710 PMC115911

[20] Epstein LG, Gendelman HE (1993) Human immunodeficiency virus type 1 infection of the nervous system: pathogenetic mechanisms. Ann Neurol 33: 429–436. doi:10.1002/ana.410330502. PubMed: 8498818. 8498818

[21] Canales RD, Luo Y, Willey JC, Austermiller B, Barbacioru CC et al. (2006) Evaluation of DNA microarray results with quantitative gene expression platforms. Nat Biotechnol 24: 1115–1122. doi:10.1038/nbt1236. PubMed: 16964225. 16964225

[22] Xue M, Shi X, Zhang J, Zhao Y, Cui H et al. (2012) Identification of a conserved B-cell epitope on reticuloendotheliosis virus envelope protein by screening a phage-displayed random peptide library. PLOS ONE 7: e49842. doi:10.1371/journal.pone.0049842. PubMed: 23185456. 23185456 PMC3504085

[23] Withanage GS, Kaiser P, Wigley P, Powers C, Mastroeni P et al. (2004) Rapid expression of chemokines and proinflammatory cytokines in newly hatched chickens infected with Salmonella enterica serovar typhimurium. Infect Immun 72: 2152–2159. doi:10.1128/IAI.72.4.2152-2159.2004. PubMed: 15039338. 15039338 PMC375210

[24] Kidd P (2003) Th1/Th2 balance: the hypothesis, its limitations, and implications for health and disease. Altern Med Rev 8: 223–246. PubMed: 12946237. 12946237

[25] Hwang ES, Szabo SJ, Schwartzberg PL, Glimcher LH (2005) T helper cell fate specified by kinase-mediated interaction of T-bet with GATA-3. Science 307: 430–433. doi:10.1126/science.1103336. PubMed: 15662016. 15662016

[26] Becker Y (2004) The changes in the T helper 1 (Th1) and T helper 2 (Th2) cytokine balance during HIV-1 infection are indicative of an allergic response to viral proteins that may be reversed by Th2 cytokine inhibitors and immune response modifiers--a review and hypothesis. Virus Genes 28: 5–18. doi:10.1023/B:VIRU.0000012260.32578.72. PubMed: 14739648. 14739648

[27] Avery S, Rothwell L, Degen WD, Schijns VE, Young J et al. (2004) Characterization of the first nonmammalian T2 cytokine gene cluster: the cluster contains functional single-copy genes for IL-3, IL-4, IL-13, and GM-CSF, a gene for IL-5 that appears to be a pseudogene, and a gene encoding another cytokinelike transcript, KK34. J Interferon Cytokine Res 24: 600–610. doi:10.1089/jir.2004.24.600. PubMed: 15626157. 15626157

[28] de Waal Malefyt R, Haanen J, Spits H, Roncarolo MG, te Velde A et al. (1991) Interleukin 10 (IL-10) and viral IL-10 strongly reduce antigen-specific human T cell proliferation by diminishing the antigen-presenting capacity of monocytes via downregulation of class II major histocompatibility complex expression. J Exp Med 174: 915–924. doi:10.1084/jem.174.4.915. PubMed: 1655948. 1655948 PMC2118975

[29] Kim R, Emi M, Tanabe K (2005) Cancer cell immune escape and tumor progression by exploitation of anti-inflammatory and pro-inflammatory responses. Cancer Biol Ther 4: 924–933. doi:10.4161/cbt.4.9.2101. PubMed: 16177562. 16177562

[30] Mocellin S, Wang E, Marincola FM (2001) Cytokines and immune response in the tumor microenvironment. J Immunother 24: 392–407. doi:10.1097/00002371-200109000-00002. PubMed: 1169669511685082. 11696695

[31] Conrad CT, Ernst NR, Dummer W, Bröcker EB, Becker JC (1999) Differential expression of transforming growth factor beta 1 and interleukin 10 in progressing and regressing areas of primary melanoma. J Exp Clin Cancer Res 18: 225–232. PubMed: 10464712. 10464712

[32] Yue FY, Dummer R, Geertsen R, Hofbauer G, Laine E et al. (1997) Interleukin-10 is a growth factor for human melanoma cells and down-regulates HLA class-I, HLA class-II and ICAM-1 molecules. Int J Cancer 71: 630–637. doi:10.1002/(SICI)1097-0215(19970516)71:4. PubMed: 9178819. 9178819

[33] Marincola FM, Jaffee EM, Hicklin DJ, Ferrone S (2000) Escape of human solid tumors from T-cell recognition: molecular mechanisms and functional significance. Adv Immunol 74: 181–273. PubMed: 10605607. 10605607 10.1016/s0065-2776(08)60911-6

[34] Moore KW, de Waal Malefyt R, Coffman RL, O'Garra A (2001) Interleukin-10 and the interleukin-10 receptor. Annu Rev Immunol 19: 683–765. doi:10.1146/annurev.immunol.19.1.683. PubMed: 11244051. 11244051

[35] Becker Y (2004) HIV-1 gp120 binding to dendritic cell receptors mobilize the virus to the lymph nodes, but the induced IL-4 synthesis by FcepsilonRI+ hematopoietic cells damages the adaptive immunity--a review, hypothesis, and implications. Virus Genes 29: 147–165. doi:10.1023/B:VIRU.0000032797.43537.d3. PubMed: 15215692. 15215692

[36] Osakwe CE, Bleotu C, Chifiriuc MC, Grancea C, Oţelea D et al. (2010) TH1/TH2 cytokine levels as an indicator for disease progression in human immunodeficiency virus type 1 infection and response to antiretroviral therapy. Roum Arch Microbiol Immunol 69: 24–34. PubMed: 21053781. 21053781

[37] Sundick RS, Gill-Dixon C (1997) A cloned chicken lymphokine homologous to both mammalian IL-2 and IL-15. J Immunol 159: 720–725. PubMed: 9218587. 9218587

[38] Lillehoj HS, Min W, Choi KD, Babu US, Burnside J et al. (2001) Molecular, cellular, and functional characterization of chicken cytokines homologous to mammalian IL-15 and IL-2. Vet Immunol Immunopathol 82: 229–244. doi:10.1016/S0165-2427(01)00360-9. PubMed: 11587737. 11587737

[39] Isaacs A, Lindenmann J (1957) Virus interference. I. Interferon - Proc R Soc Lond B Biol Sci 147: 258–267. doi:10.1098/rspb.1957.0048. 26297790

[40] Ortegel JW, Staren ED, Faber LP, Warren WH, Braun DP (2002) Modulation of tumor-infiltrating lymphocyte cytolytic activity against human non-small cell lung cancer. Lung Cancer 36: 17–25. doi:10.1016/S0169-5002(01)00472-X. PubMed: 11891029. 11891029

[41] Lüscher U, Filgueira L, Juretic A, Zuber M, Lüscher NJ et al. (1994) The pattern of cytokine gene expression in freshly excised human metastatic melanoma suggests a state of reversible anergy of tumor-infiltrating lymphocytes. Int J Cancer 57: 612–619. doi:10.1002/ijc.2910570428. PubMed: 8181865. 8181865

[42] Jarosinski KW, Njaa BL, O'Connell PH, Schat KA (2005) Pro-inflammatory responses in chicken spleen and brain tissues after infection with very virulent plus Marek's disease virus. Viral Immunol 18: 148–161. doi:10.1089/vim.2005.18.148. PubMed: 15802959. 15802959

[43] Dinarello CA, Fantuzzi G (2003) Interleukin-18 and host defense against infection. J Infect Dis 187 Suppl 2: S370–S384. doi:10.1086/374751. PubMed: 12792854. 12792854

[44] Metcalf D (1989) The molecular control of cell division, differentiation commitment and maturation in haemopoietic cells. Nature 339: 27–30. doi:10.1038/339027a0. PubMed: 2469962. 2469962

[45] Hamilton JA (1993) Rheumatoid arthritis: opposing actions of haemopoietic growth factors and slow-acting anti-rheumatic drugs. Lancet 342: 536–539. doi:10.1016/0140-6736(93)91653-4. PubMed: 8102674. 8102674

[46] Aukrust P, Liabakk NB, Müller F, Lien E, Espevik T et al. (1994) Serum levels of tumor necrosis factor-alpha (TNF alpha) and soluble TNF receptors in human immunodeficiency virus type 1 infection--correlations to clinical, immunologic, and virologic parameters. J Infect Dis 169: 420–424. doi:10.1093/infdis/169.2.420. PubMed: 7906293. 7906293

[47] Calzascia T, Pellegrini M, Hall H, Sabbagh L, Ono N et al. (2007) TNF-alpha is critical for antitumor but not antiviral T cell immunity in mice. J Clin Invest 117: 3833–3845. PubMed: 17992258. 17992258 10.1172/JCI32567 PMC2066188

[48] Ebnet K, Vestweber D (1999) Molecular mechanisms that control leukocyte extravasation: the selectins and the chemokines. Histochem Cell Biol 112: 1–23. doi:10.1007/s004180050387. PubMed: 10461808. 10461808

[49] Murtaugh MP, Baarsch MJ, Zhou Y, Scamurra RW, Lin G (1996) Inflammatory cytokines in animal health and disease. Vet Immunol Immunopathol 54: 45–55. doi:10.1016/S0165-2427(96)05698-X. PubMed: 8988847. 8988847

[50] Baggiolini M, Dewald B, Moser B (1997) Human chemokines: an update. Annu Rev Immunol 15: 675–705. doi:10.1146/annurev.immunol.15.1.675. PubMed: 9143704. 9143704

[51] Khatri M, Sharma JM (2006) Infectious bursal disease virus infection induces macrophage activation via p38 MAPK and NF-kappaB pathways. Virus Res 118: 70–77. doi:10.1016/j.virusres.2005.11.015. PubMed: 16388870. 16388870

[52] Balkwill F, Mantovani A (2001) Inflammation and cancer: back to Virchow? Lancet 357: 539–545. doi:10.1016/S0140-6736(00)04046-0. PubMed: 11229684. 11229684

[53] Knudsen BS, Allen AN, McLerran DF, Vessella RL, Karademos J et al. (2008) Evaluation of the branched-chain DNA assay for measurement of RNA in formalin-fixed tissues. J Mol Diagn 10: 169–176. doi:10.2353/jmoldx.2008.070127. PubMed: 18276773. 18276773 PMC2259472

